# Meeting the Challenge of Teaching Information Literacy

**DOI:** 10.29173/jchla29574

**Published:** 2021-08-01

**Authors:** Megan Kennedy

**Affiliations:** Public Services Librarian, University of Alberta, Edmonton, AB, Canada

Reale M. **Meeting the Challenge of Teaching Information Literacy**. 1st ed. Chicago: ALA Editions; 2020. Softcover: 112p. ISBN: 978-0-8389-4684-8. Price: USD $57.99. Available from: https://www.alastore.ala.org/content/meeting-challenge-teaching-information-literacy

In this book, Michelle Reale gets to the core of *why* information literacy (IL) is so difficult for librarians to teach. Situating her work in the current literature, Reale comments that there is a large body of literature that highlights best practices for teaching IL, why it is an important element of an academic librarian's work and why we are best positioned to deliver this type of content. Despite this extensive existing literature, Reale notes that little is truly known about why we struggle to effectively deliver this content to students and faculty. I found this text to be thought provoking and it made me think more purposefully about the ways in which I deliver IL instruction to students and faculty. Reale admits that this is not a quick fix guide to solving the problems associated with IL instruction and the text offers little in the way of solid solutions. Rather, Reale states that “these chapters seek to explicate a problem or issue that we can further reflect on to find individual solutions” [page viii]. Further, this work “is meant to highlight areas of challenge and concern to those of us who teach IL in academia. It is meant to be used, and, in fact, is best used, in conjunction with reflective practice as a way of understanding the origins of our challenges, which then allows us to forge a path forward” [page ix].

Michelle Reale is a professor and access services and outreach librarian at Arcadia University, a private university in Pennsylvania, USA. She has written several books on the topics of instruction as an academic librarian, embedded librarianship, and reflective practice. Reale holds three Master's degrees in English, Library Science, and Creative Writing (Poetry). I think that Reale's dedication to creative writing can be seen in the text as the chapters are written in a very approachable and colloquial manner with very little jargon and great personal stories. Reale notes that this informal tone is intentional so as not to create “theoretical distance”.

Reale pulls from hers and colleagues' own real-life experiences delivering IL instruction and provides personal anecdotes to set up the themes for each of the ten chapters. The chapters begin with a short scenario that is then dissected in the main text of the chapter. Reale finishes each chapter with “Points to Ponder” which are thought provoking questions meant to help the reader reflect on their own teaching practices. Reale succinctly highlights known issues for librarians teaching IL. Some of the highlights and challenges I most resonated with were: challenges engaging students (and at times, faculty), misaligned or poorly considered timing and placement of IL instruction in the curriculum or course, disengaged instructors who are simply “checking the box” to have a librarian deliver instruction in their classroom, lack of support at the institutional level for librarians to develop instruction skills, piecemeal and disjointed IL instruction that comes with “one-shot” instruction sessions, and the struggle to have outsiders understand the work of librarians. Particularly interesting, Reale mentions the “library identity crisis” as many of us “combat a shifting identity that has made us in the profession prone to believing that we must justify the very existence of the profession, our libraries, and by natural extension, ourselves” [page 3]. She notes that many of us are really just striving for validation that what we do is important and useful. I found this last point to be very interesting, although I do not wholeheartedly agree with the statement. Reale later discusses common “librarian stereotypes” and the way in which these have dogged the profession, leading to librarianship being “feminized, scrutinized, debased, misunderstood, and devalued” [page 29]. I found this addition to the discussion to be very interesting as this is not something that comes to the top of my mind when I think about the challenges of IL instruction.

Overall, I think that any academic librarian who delivers IL instruction could find this text useful. Further, despite not being explicitly aimed at early career librarians, this work may be of particular interest to librarians who are just getting their feet wet with IL instruction as this book does a good job of highlighting some of the potential pitfalls related to our work as instructors. Reale's style of writing is entertaining and accessible and the examples of IL instruction challenges are relatable, well presented and well referenced (although the text is not heavy with references). However, if readers are looking for quick solutions or firm advice to overcome the issues raised, then this is not the most useful book as it is intended to generate discussion and self-reflection on teaching practice.

